# VE-Cadherin-Independent Cancer Cell Incorporation into the Vascular Endothelium Precedes Transmigration

**DOI:** 10.1371/journal.pone.0109748

**Published:** 2014-10-02

**Authors:** Susan M. Hamilla, Kimberly M. Stroka, Helim Aranda-Espinoza

**Affiliations:** Fischell Department of Bioengineering, University of Maryland, College Park, Maryland, United States of America; Vanderbilt University, United States of America

## Abstract

Metastasis is accountable for 90% of cancer deaths. During metastasis, tumor cells break away from the primary tumor, enter the blood and the lymph vessels, and use them as highways to travel to distant sites in the body to form secondary tumors. Cancer cell migration through the endothelium and into the basement membrane represents a critical step in the metastatic cascade, yet it is not well understood. This process is well characterized for immune cells that routinely transmigrate through the endothelium to sites of infection, inflammation, or injury. Previous studies with leukocytes have demonstrated that this step depends heavily on the activation status of the endothelium and subendothelial substrate stiffness. Here, we used a previously established *in vitro* model of the endothelium and live cell imaging, in order to observe cancer cell transmigration and compare this process to leukocytes. Interestingly, cancer cell transmigration includes an additional step, which we term ‘incorporation’, into the endothelial cell (EC) monolayer. During this phase, cancer cells physically displace ECs, leading to the dislocation of EC VE-cadherin away from EC junctions bordering cancer cells, and spread into the monolayer. In some cases, ECs completely detach from the matrix. Furthermore, cancer cell incorporation occurs independently of the activation status and the subendothelial substrate stiffness for breast cancer and melanoma cells, a notable difference from the process by which leukocytes transmigrate. Meanwhile, pancreatic cancer cell incorporation was dependent on the activation status of the endothelium and changed on very stiff subendothelial substrates. Collectively, our results provide mechanistic insights into tumor cell extravasation and demonstrate that incorporation is one of the earliest steps.

## Introduction

Cancer metastasis occurs when tumor cells fragment from the primary tumor site, enter the blood and lymph vessels, and spread to distant bodily organs. This process is one of the main contributing factors to the deadliness of cancer [1], [2]. Once metastatic cancer cells have entered the blood stream, they must cross the endothelial cell (EC) barrier before invading the tissue beneath in a step known as extravasation. Most tumor cells arrest by nonspecific binding of coagulation factors and by size restriction in capillary beds [3]. In some cases, specific ligands on tumor cells have been correlated with an increased metastatic potential [4]–[6]. Thus far, significant research has been dedicated to analyzing the biochemical and molecular capabilities of cancer cells [7]–[9], but the underlying mechanism of cancer cell extravasation through the endothelium remains largely unknown. Cancer cells have been observed to migrate through the EC cell body [10], and through endothelial cell-cell junctions without destroying the EC layer [11]. However, conflicting research has also shown that cancer cells do not leave the endothelium intact following extravasation [10], [12]–[14]. It is important to note that these studies used different tumor cell lines, as well of different EC lines and *in vitro* methods, so it is possible that different combinations of various types of tumor cells and ECs may lead to diverging mechanisms of extravasation. There are three proposed methods of cancer cell migration through the endothelium: (a) cancer cells may migrate through the EC body [10], (b) cancer cells may induce EC apoptosis [10], [13] and (c) cancer cells may migrate through endothelial cell-cell junctions without permanently destroying the EC layer [11]. In recent years, research has also shown that cancer cells also exert forces on ECs that push them deeper into the extracellular matrix during transmigration [15], [16], and that the endothelium enhances cancer cell migration [17]. These findings suggest that cancer migration through the endothelium is a complex process that requires further investigation to elucidate its mechanistic course.

Leukocytes routinely transmigrate through the endothelium and underlying layers of the vasculature to reach tissue sites of inflammation, infection, or injury. This is a well-characterized process that relies on localized biochemical signals. In leukocyte trafficking, the endothelium acts as a selective barrier that greatly reduces the invasion rate [18]. During an immune response, the chemokine tumor necrosis factor alpha (TNF-α) is produced by stromal cells, and the localized exposure of ECs to TNF-α upregulates adhesion molecules such as intercellular adhesion molecule-1 (ICAM-1) on the surface of the endothelium. Furthermore, in addition to molecular changes, TNF-α also significantly alters the structural properties of the endothelium, which induces softening, actin realignment, and an increase in overall permeability [19], [20]. An additional factor that enhances leukocyte transmigration is the subendothelial substrate stiffness and mechanical properties of the endothelium. These vary during vascular homeostasis and in pathological conditions [19]. Neutrophils are able to mechanosense their microenvironment [19], [21]–[24], and neutrophil transmigration increases as subendothelial substrate stiffness increases due to EC myosin light chain kinase (MLCK)-mediated contractile forces [19]. All of these changes in the EC monolayer facilitate leukocyte transmigration during an immune response.

Since leukocytes routinely transmigrate through the EC layer, it is presumed that metastatic cancer cells may share many of the same mechanisms. However, these mechanisms have yet to be investigated in greater detail. For example, the involvement of chemokines in tumor-endothelial interactions and their effects on cancer cell migration are not well understood [25]. Even more, the significant molecular and structural changes that take place in the endothelium following TNF-α treatment may enhance metastatic cancer cell transmigration. It also remains to be seen how cancer cell extravasation varies with subendothelial substrate stiffness. As observed with leukocytes [19], it is possible that cancer cell transmigration may increase with increasing substrate stiffness. Cancer cells have also been observed to push ECs into the extracellular matrix during extravasation, indicating that the mechanical properties of the ECM may play an important role. Taken together, these results indicate that both the activation status of the endothelium and subendothelial substrate stiffness may influence cancer cell extravasation.

In this work, an *in vitro* model of the vascular endothelium [19], [26]–[28] was used to explore cancer cell transmigration and how this process compares to leukocyte extravasation. Our results show that one of the earliest steps in the extravasation of metastatic breast cancer cells is incorporation into the EC monolayer, which significantly disrupts the EC barrier and physically displaces ECs, sometimes leading to complete removal of ECs from the monolayer. Unlike leukocyte transmigration, cancer cell incorporation does not depend on the activation status of the endothelium or subendothelial substrate stiffness for melanoma cells and breast cancer cells. Interestingly, pancreatic cell incorporation depends on the activation status of the endothelium and the subendothelial stiffness. Together, our results indicate that metastatic breast cancer cell extravasation involves an additional step of incorporation into the endothelium, which does not occur during leukocyte extravasation.

## Materials and Methods

### Preparation of polyacrylamide gel substrates

Thin polyacrylamide gels were prepared on glass coverslips according to the method first described by Wang and Pelham [29] and described in detail in our previous publications [19], [23], [30]–[33]. Briefly, 280 kPa (15% acrylamide + 1.2% bis acrylamide) and 0.87 kPa (3% acrylamide + 0.1% bis acrylamide) gels were created and coated with 0.1 mg/mL fibronectin (Sigma-Aldrich) as previously described [19]. Characterization of the Young's modulus of the gels was accomplished by atomic force microscopy and dynamic mechanical analysis, while analysis of surface-bound fibronectin was accomplished by immunofluorescence [23], [32]. For experiments on glass, 22×22 mm coverslips (Fisher Scientific) were coated with 0.1 mg/mL fibronectin for 2 hours at room temperature.

### Cell culture

Human umbilical vein endothelial cells (Lifeline Cell Technology) were cultured as previously described [34]. MDA-MB-231 metastatic breast cancer cells (ATCC) and A375 melanoma cells (ATCC) were cultured in DMEM, 10% FBS and 1% penicillin streptomycin. SW1990 pancreatic cancer cells (ATCC) were cultured in DMEM, 10% FBS, and 50 µg/mL gentamicin. HUVECs (passages 2–5, 4×10^5^ total) were plated onto fibronectin-coated glass coverslips or polyacrylamide gels and grown for approximately 48 hours, at which point a monolayer formed. Cells were treated with control media or 25 ng/mL tumor necrosis factor-α (TNF-α) for the final 24 hours prior to experiments. In some experiments, HUVECs were transfected with VE-cadherin-GFP (VEcadGFP) using an adenovirus (AdV), which was received as a generous gift from Dr. William Luscinskas (Harvard Medical School). Dr. Luscinskas's lab has previously described the procedure for construction of the VEcadGFP plasmid and transference to an adenovirus expression vector [35]. HUVECs were plated onto fibronectin-coated polyacrylamide gels or glass coverslips and given 1–2 hours to spread, and 3 µL of AdV-VEcadGFP were added to the cells with 2 mL media per substrate. HUVECs were then cultured to monolayer formation as described above. Monolayers were washed with PBS prior to adding additional HUVECs or tumor cells (1×10^5^ cells total) to the apical surface of the 22×22 mm monolayer. To distinguish between the HUVEC cells within the monolayer and the additional cells added to the monolayer, added HUVECs or MDA-MB-231 cells were stained with the lipophilic DiIC_16_ dye (1 µM) in suspension for 5 minutes at room temperature, followed by centrifugation and a PBS wash.

### Live cell imaging and analysis

Live cell microscopy was completed in an enclosed microscope stage incubator at 37°C, 5% CO_2_ and 55% humidity using an inverted microscope (Olympus IX71). Images were captured with either a QImaging Retiga-SRV charge-coupled device (CCD) digital camera (QImaging Corporation) using IPLab software (Becton, Dickinson and Company), or with a a QImaging Rolera-MGi CCD digital camera (QImaging Corporation) using Slidebook software (version 4.2.0.9; Intelligent Imaging Innovations). Phase contrast, differential interference contrast (DIC), and/or fluorescence timelapse images were captured using either a 20×/0.45 NA Ph1 objective or a 60×/1.42 NA oil objective. The fraction of incorporated cells (HUVECs or MDA-MB-231) was calculated by dividing the number of cells that became incorporated at any time during the timelapse sequence by the total number of phase-white cells above the monolayer in the initial frame of the sequence. The time to complete incorporation was calculated using the difference between the time when the cell first began to spread into the monolayer and the time when the cell reached maximum spreading area within the monolayer.

### Interference reflection microscopy

Interference reflection microscopy (IRM) was used to detect surface-to-surface interference between light rays reflected from the substrate/medium interface and those from the medium/cell interface, as described in our previous work [36]–[38]. In this technique, the intensity of the light is a measure of the proximity of the cell to the glass surface, such that areas of the membrane closest to the surface appear dark and those further away appear brighter. Therefore, IRM is an optimal method when evaluating cellular attachment, adhesion, and spreading behavior [36]–[38]. For spreading experiments, 1×10^5^ MDA-MB-231 cells were plated onto 22×22 mm fibronectin-coated glass coverslips in HUVEC media, resulting in observation of single cells. For these experiments, an inverted microscope (Olympus IX71) with a 60×/1.42 NA oil objective lens and a 100 W mercury lamp (Olympus; used at wavelength 561) was used in combination with a CCD camera (Retiga SRV camera, QImaging) for image capture. Experiments were performed in an enclosed microscope chamber which maintained culture conditions at 37°C, 50% humidity, and 5% CO_2._ During cell spreading, one frame was recorded every 5 seconds over a period of 1 µhour for N = 5 independent experiments. For statistical evaluations of spreading areas, images were analyzed using ImageJ (National Institutes of Health) software. The cell-boundaries were traced by hand and area was calculated using ImageJ routines. Areas of MDA-MB-231 cells spreading into a HUVEC monolayer were also traced by hand and compared with single cells spreading onto the endothelium-free coverslip.

### Confocal Imaging

A total of 1×10^5^ MDA-MB-231 cells were transfected with 10µL of CellLight Actin-GFP (Life Technologies) according to manufacturer's protocol and allowed to incubate for 24 hours. After 24 hours, infected MDA-MB-231 cells were introduced to a confluent HUVEC monolayer and allowed to incorporate for 15 hours. Following incorporation, cells were fixed in 4% paraformaldehyde and stained using Texas-Red Phalloidin (Invitrogen). Z-stack images were collected using a Zeiss LSM 710 confocal microscope with a 63× oil objective.

### Statistical analysis

Statistical analysis was completed by using a Student t-test between pairs of data, or by using ANOVA for groups of data, where P<0.05 indicated statistical significance. After ANOVA, multiple comparisons were done using Turkey's honestly significant difference criterion. All measurements are reported here in the format mean ± standard error.

## Results

### Incorporation into the endothelium is the first step in metastatic cancer cell transmigration

**Figure 1:**
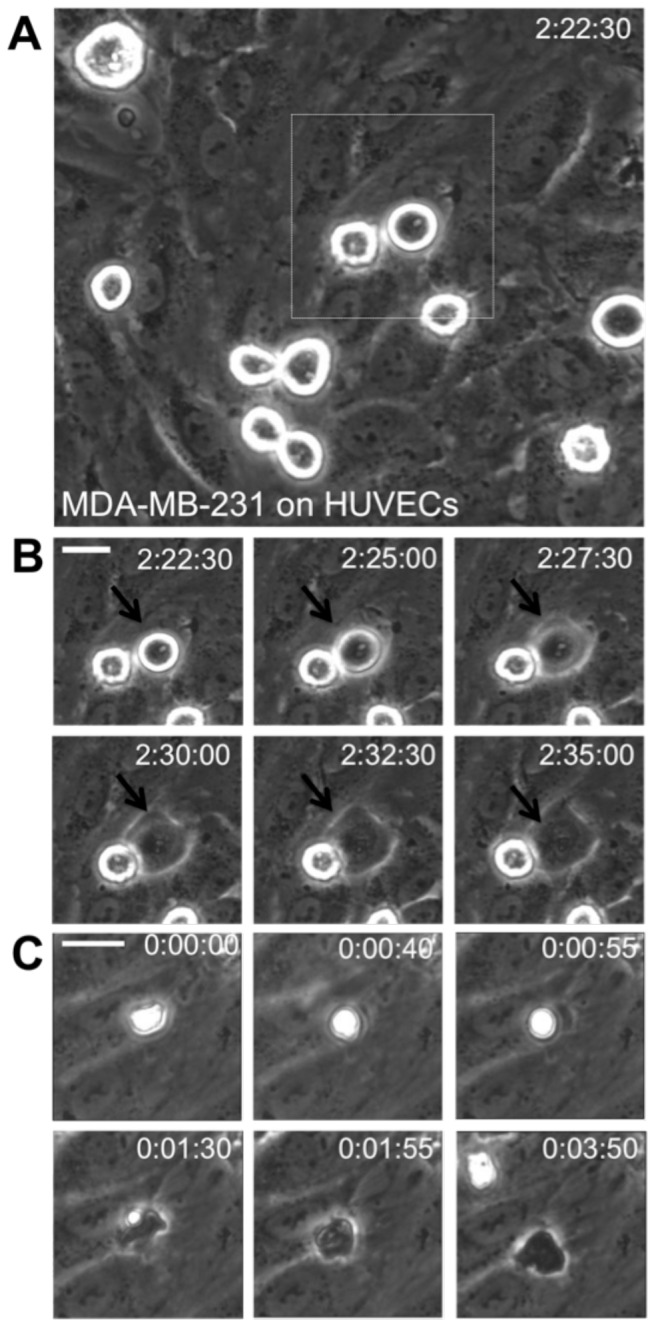
Incorporation of MDA-MB-231 metastatic breast cancer cells is a first step in the extravasation process. (A) Phase contrast image of MDA-MB-231 cells (bright white) atop a human umbilical vein endothelial (HUVEC) monolayer. Scale bar is 20 µm. (B) MDA-MB-231 cell (black arrows) begins to incorporate into the endothelium, as indicated by its change in phase contrast microscopy from bright white to darkened. Scale bar is 20 µm and applies to all images in panel B. Length of time after plating MDA-MB-231 cells on the endothelium is indicated in the upper right corner of each image in hour:minute:second format. The final percentage of incorporation for this experiment was 95%. (C) Phase contrast image sequence of a neutrophil transmigrating through a TNF-α-activated endothelium. Scale bar is 20 µm and applies to all images in panel C. Length of time after plating neutrophils on the endothelium is indicated in the upper right corner of each image in hour:minute:second format.

**Figure 2:**
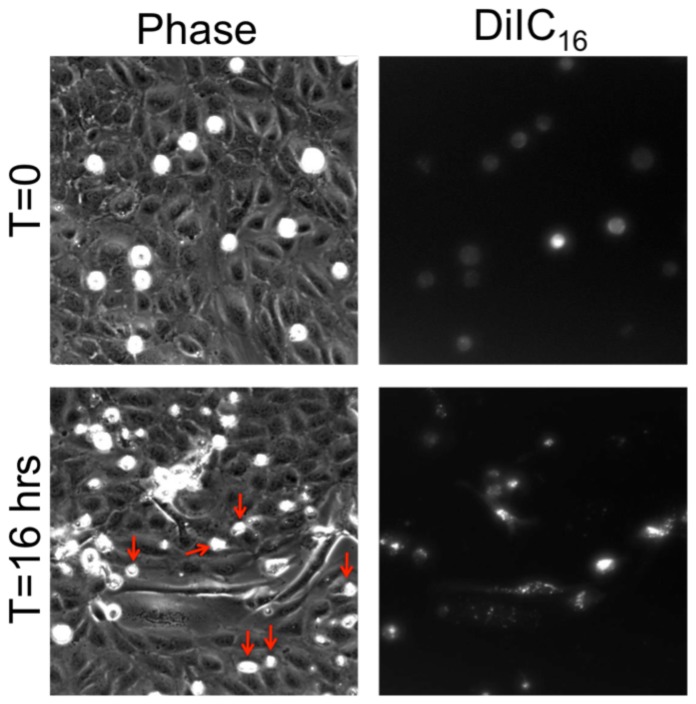
MDA-MB-231 incorporation causes detachment and rounding of some endothelial cells. Phase contrast (left) and DiIC_16_ fluorescence (right) images of MDA-MB-231 cells plated onto an untreated HUVEC monolayer, at time points immediately after plating (top) and after 16 hours of interaction with the endothelium (bottom). Red arrows point to phase-white cells that do not emit fluorescence; these are endothelial cells that have been forced out of the monolayer and thus have detached and become rounded. Scale bar is 25 µm and applies to all images.

**Figure 3:**
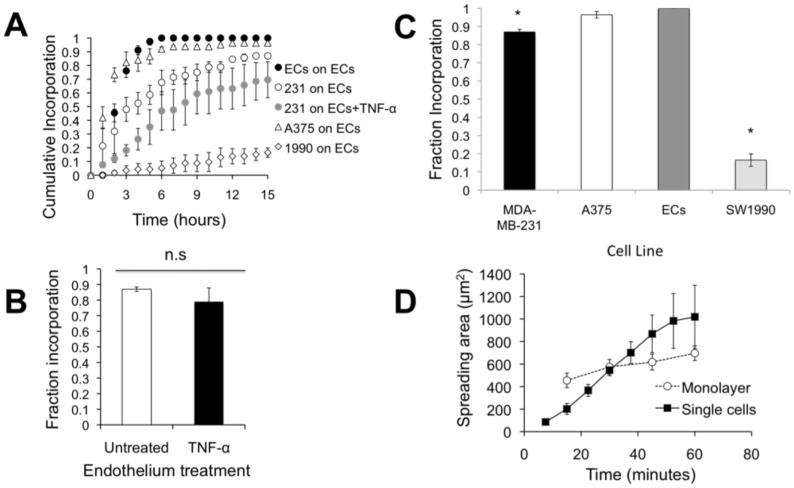
Incorporation of MDA-MB-231 does not depend on whether the endothelium is activated by TNF-α. (A) Cumulative fraction of ECs or MDA-MB-231 cells (231), SW1990 (1990), and A375 cells incorporated into the endothelium as a function of time after plating. Data points represent mean ± SEM for at least 3 independent experiments (N>20 cells for each experiment). (B) Final fraction of MDA-MB-231 cells incorporated into the untreated or TNF-α-treated endothelium after 15 hours. Bars represent mean, while error bars represent SEM of at least 3 independent experiments. P>0.05 between these values indicates there is no statistical difference (n.s.). (C) Final fraction of MDA-MB-231 breast cancer cells, ECs, A375 melanoma cells, and SW1990 pancreatic cells incorporated into the endothelium after 15 hours. Bars represent mean, while error bars represent SEM of at least 3 independent experiments. (*) indicates significance (P<0.05) when compared to ECs. (D) Plot of spreading area versus time reveals differences in spreading dynamics for MDA-MB-231 cells spreading onto a fibronectin-coated coverslip (“single cells”) or into an untreated endothelium (“into monolayer”).

**Figure 4:**
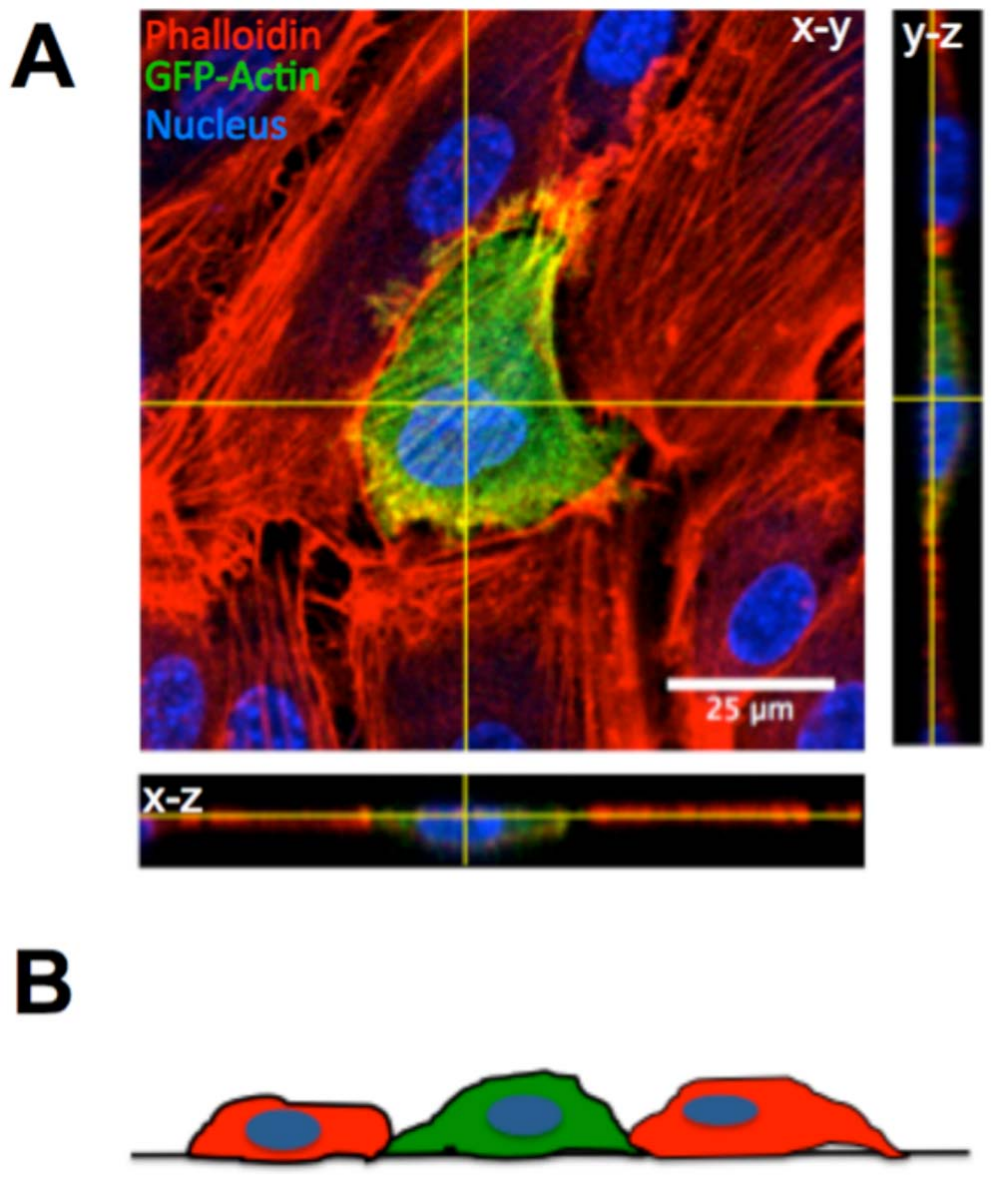
Confocal images reveal that MDA-MB-231 cells do not migrate underneath ECs during the incorporation process. (A) A representative MDA-MB-231 (green; Actin-GFP) cell infected with GFP-actin is shown spreading into a HUVEC monolayer (red; Phalloidin). Orthogonal projections are shown. (B) Schematic showing that a cancer cell (green) displaces ECs (red) by spreading between adjacent ECs during incorporation.

MDA-MB-231 metastatic breast cancer cells were introduced onto the apical surface of an EC monolayer and monitored as they interacted with the endothelium (Movie S1). Initially, MDA-MB-231 cells were bright white and spherical in contrast to the underlying phase-darkened and flattened endothelium (Fig. 1A). Within several hours, the MDA-MB-231 cells began to incorporate into the endothelium, in a process that ranged on average from 40 to 60 minutes (Fig. 1B) and was visually distinct from neutrophil transmigration through the endothelium (Fig. 1C). Specifically, it appeared that the ECs adjacent to the site of incorporation were physically displaced, creating voids for the MDA-MB-231 cell to spread onto the underlying matrix (Fig. 1A), whereas neutrophils squeezed through the endothelium without disrupting the monolayer (Fig. 1C). Some ECs detached from the underlying matrix, rounded up and became spherical following incorporation of MDA-MB-231 cells into the monolayer (Fig. 2). Approximately 25% of the ECs detached following cancer cell incorporation (Fig 2). However, incorporation of MDA-MB-231 cells into the endothelium occurred more slowly, with a smaller slope of “spreading area vs. time”, and had a lower final cellular area compared to MDA-MB-231 cells spreading onto an endothelium-free fibronectin-coated substrate (Fig. 3D), indicating that the endothelium did not favor incorporation of the cancer cell, but rather limited overall spreading area. Furthermore, MDA-MB-231 cell incorporation into the endothelium occurred more slowly, with a smaller slope of “cumulative incorporation vs. time”, in comparison to ECs incorporating into a monolayer of ECs (Fig. 3A). Thus, it is likely that incorporation of MDA-MB-231 cells into the endothelium occurs by a different mechanism than native ECs spreading into the same endothelium. A375 melanoma cells and SW1990 pancreatic cells were also introduced to the apical surface of the endothelium. Similarly to MDA-MB-231 cells, the melanoma cells and pancreatic cells began to incorporate into the endothelium in a process that lasted approximately 15 minutes and 50 minutes respectively. A375 cancer cells incorporated into the monolayer at a much faster rate compared to metastatic breast cancer cells and their incorporation dynamics closely resembled that of native ECs spreading into the EC monolayer. A375 melanoma cells had a final fraction of incorporation that resembled ECs spreading into ECs, while SW1990 cancer cells had a much lower fraction of incorporation compared to all groups (Fig 3C). Furthermore, confocal images (Fig 4A) revealed that after 15 hours of incorporation, MDA-MB-231 (green) cells displaced ECs (red) by spreading between adjacent ECs. Orthogonal projections show that MDA-MB-231 cells are in fact spreading between ECs and not migrating underneath them during incorporation (Fig 4B).

### Activation of the endothelium by TNF-α does not affect incorporation dynamics of breast cancer and melanoma cells

**Figure 5:**
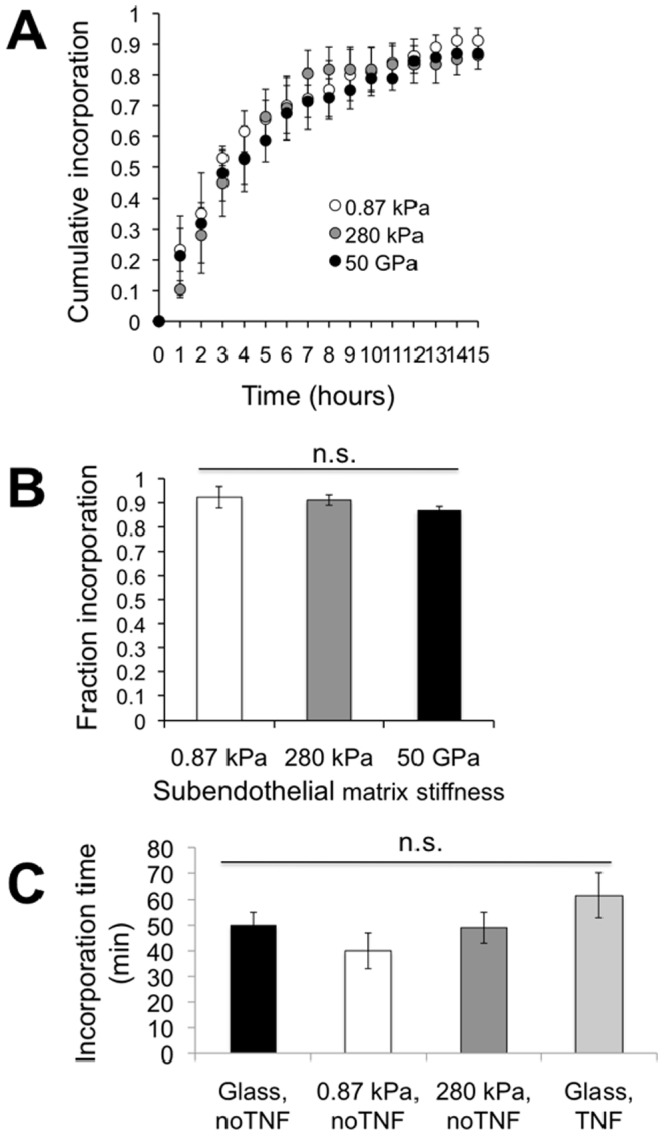
Incorporation of MDA-MB-231 does not depend on subendothelial substrate stiffness. (A) Cumulative fraction of MDA-MB-231 cells incorporated into endothelial cells on a fibronectin-coated 0.87 kPa or 280 kPa polyacrylamide gel, or glass (50 GPa). Data points represent mean ± SEM for at least 3 independent experiments (N>20 cells for each experiment). (B) Final fraction of MDA-MB-231 cells incorporated into the (untreated) endothelium as a function of subendothelial substrate stiffness. Bars represent mean, while error bars represent SEM of at least 3 independent experiments. P>0.05 between these values indicates there is no statistical difference (n.s.). (C) Time for MDA-MB-231 cells to complete incorporation is independent of the mechanical properties of the substrate below the endothelial cells. Endothelial cells on fibronectin-coated glass coverslips (50 GPa) or polyacrylamide gels (0.87 kPa or 280 kPa) were left untreated (no TNF) or treated with TNF-α (TNF). No statistical difference in incorporation time was measured as a function of subendothelial substrate stiffness or endothelial cell treatment (P>0.05).

Activation of the endothelium is a key step in the leukocyte adhesion cascade, and our previous work has demonstrated that leukocyte transmigration depends heavily on the activation status of the endothelium [19], [28], [30]. Activation of the endothelium by TNF-α not only upregulates adhesion molecules such as ICAM-1 and vascular cell adhesion molecule-1 (VCAM-1), but also increases EC contractility [31] and reduces EC barrier function [20]. Therefore, our next goal was to determine whether incorporation of metastatic breast cancer cells into the endothelium required the ECs to be activated. Intriguingly, we found that the cumulative fraction of incorporation versus time of MDA-MB-231 cells was similar, regardless of whether the ECs were treated with TNF-α (Fig. 3A). In addition, the final fraction of cells that incorporated into a TNF-α-activated endothelium after 15 hours was not different from the fraction incorporated into an untreated endothelium (Fig. 3B). Finally, the time required to complete incorporation (from a rounded sphere to a flattened cell within the endothelium) was not dependent on whether the endothelium was treated with TNF-α (Fig. 5C). Similar to breast cancer cells, the fraction of incorporation of A375 melanoma cells into ECs was comparable, regardless of whether the ECs were treated with TNF-α (Fig S1). These results suggest that some metastatic cancer cells are able to complete the early stages of extravasation, even in the absence of an inflammatory stimulus. However, the fraction of incorporation after 15 hours for SW1990 pancreatic cells was statistically different between untreated ECs and those treated with TNF-α (Fig S2). SW1990 cells incorporated at a much faster rate and higher fraction in TNF-α treated ECs compared to untreated ECs.

### Incorporation is independent of subendothelial substrate stiffness for breast cancer and melanoma cells

Our previous work has also demonstrated that leukocyte transmigration depends on the mechanical properties of the substrate below the endothelial cells [19], [30]. Stiffer subendothelial matrices promote myosin light chain kinase dependent EC contractility, leading to intercellular gaps and enhanced leukocyte transmigration [19], [30]. Therefore, our next goal was to establish whether metastatic breast cancer cell incorporation into the endothelium was dependent on subendothelial substrate stiffness. Unlike neutrophil transmigration, which increases with subendothelial substrate stiffness, the dynamics of MDA-MB-231 cell incorporation into the endothelium were similar for soft (0.87 kPa), intermediate (280 kPa), and very stiff (glass; 50 GPa) subendothelial substrates (Fig. 5A). In addition, the final incorporated fraction of MDA-MB-231 into the endothelium was independent of subendothelial substrate stiffness (Fig. 3B). and the total time required to complete transmigration was independent of subendothelial substrate stiffness (Fig. 5C). A375 melanoma cells incorporated into soft, intermediate, and very stiff subendothelial matrices with similar incorporation dynamics and final fraction of incorporation to breast cancer cells (Fig S3). SW1990 pancreatic cells displayed a different behavior from the melanoma cells and breast cancer cells (Fig S4). When SW1990 cells were plated on soft and intermediate substrates, they displayed a similar level of incorporation dynamics and final fraction of incorporation after 15 hours (Fig S4). However, when allowed to incorporate on very stiff matrices (glass; 50 GPa), they displayed a much lower fraction of incorporation and slower incorporation rate. While subsequent steps of the metastatic cascade may depend on substrate stiffness, our results indicate that the early stages of breast cancer cell extravasation and melanoma extravasation occur irrespective of the mechanical properties of the matrix below the endothelium while pancreatic cell incorporation changes on very stiff substrates.

### Endothelial cells do not express VE-cadherin along borders with incorporated breast cancer cells

**Figure 6:**
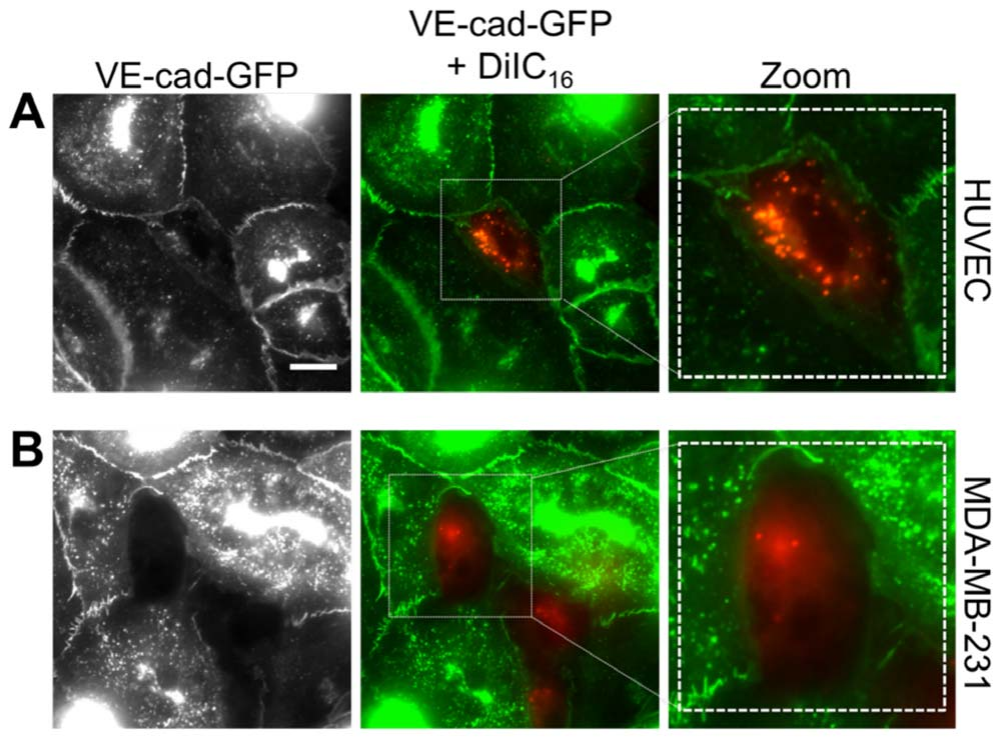
Endothelial cells do not express VE-cadherin along borders with incorporated cancer cells. DiIC16-labeled HUVECs (A) or MDA-MB-231 (B) were plated onto endothelial cells expressing VE-cadherin-GFP (VE-cad-GFP). Images were captured following incorporation of each cell type. Scale bar is 10 µm and applies to all images in this figure.

Vascular endothelial cadherin (VE-cadherin) is one of the key homophilic adhesion molecules expressed by ECs at cell-cell borders in a confluent monolayer. The integrity of the endothelium as a vascular barrier is heavily dependent on the proper functioning of VE-cadherin adhesion molecules [39], [40]. As ECs incorporated into a healthy confluent endothelium, GFP-VE-cadherin was expressed by all ECs neighboring the incorporated DiIC_16_-labeled EC (Fig. 6A, Movie S2), indicating that addition of new ECs onto the monolayer did not disrupt the integrity of the EC junctions. We next sought to identify possible changes in VE-cadherin morphology following incorporation of MDA-MB-231 cells into the endothelium. Strikingly, ECs neighboring incorporated DiIC_16_-labeled MDA-MB-231 cells did not express GFP-VE-cadherin at borderlines with the MDA-MB-231 cells, though they continued to express GFP-VE-cadherin at junctions with other ECs (Fig. 6B). These results indicate that the early stage of cancer cell extravasation not only affects VE-cadherin-dependent cell-cell adhesion, but may also provide an easily accessible route of extravasation for other circulating tumor cells.

### Breast cancer cell incorporation initiates by dislocating VE-cadherin at endothelial cell junctions

**Figure 7:**
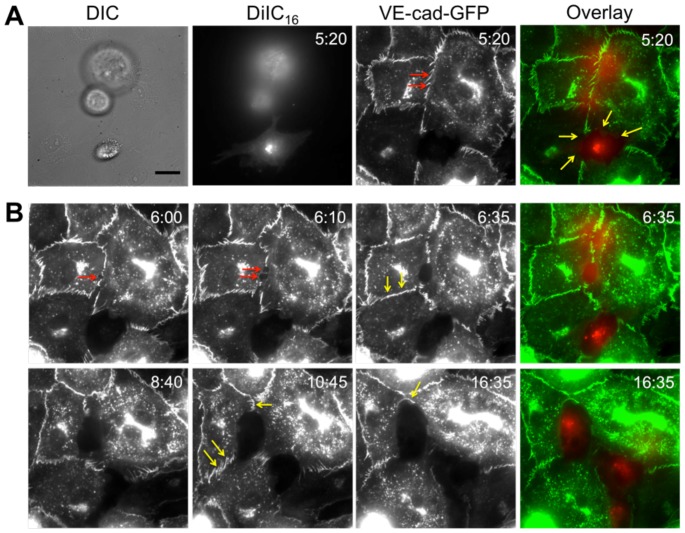
Cancer cell incorporation initiates by dislocating VE-cadherin at endothelial cell junctions. DiIC_16_-labeled MDA-MB-231 cells were plated onto endothelial cells expressing VE-cadherin-GFP (VE-cad-GFP). (A) Shown are differential interference contrast (DIC), DiIC_16_ (red) fluorescence, and VE-cadherin-GFP (green) fluorescence, and overlay images. At this time point, one MDA-MB-231 has already incorporated into the endothelium (yellow arrows), and the VE-cadherin-GFP is still intact in the location directly below another MDA-MB-231 cell that has not yet begun to incorporate (red arrows). (B) Fluorescence timelapse sequence of a DiIC_16_-labeled MDA-MB-231 cell (red) incorporating into an endothelium expressing VE-cadherin-GFP (green). Length of time after plating MDA-MB-231 cells on the endothelium is indicated in the upper right corner of each image in hour:minute format. Scale bar in panel A (DIC image) is 10 µm and applies to all images in this figure.

Prior to the onset of MDA-MB-231 incorporation into the endothelium, we observed intact GFP-VE-cadherin at the EC junction directly below the MDA-MB-231 cell (Fig. 7A; red arrows, Movie S2). This was in direct contrast to the MDA-MB-231 cells that incorporated into the endothelium at earlier time points, where GFP-VE-cadherin was not expressed by neighboring ECs (Fig. 7A; yellow arrows). The first step of incorporation created a disruption in GFP-VE-cadherin at the EC junction directly below the MDA-MB-231 cell, leading to formation of a small (∼2 µm^2^) hole in the EC junction (Fig. 7B; red arrow). A second small hole sometimes formed (Fig. 5B; two red arrows) before the void became significantly larger (∼33 µm^2^ at T = 6∶35) and cleared the area of part of the body of the EC. Finally, what began as a small gap in the GFP-VE-cadherin propagated into a huge hole in the endothelium, which became occupied by the MDA-MB-231 cell (Fig. 7B). We also observed significant displacement and restructuring of GFP-VE-cadherin at nearby EC-EC junctions (Fig. 7B; yellow arrows).

## Discussion

Cancer metastasis continues to be a leading cause of death in cancer patients [1], [2], and one of the critical steps regulating metastasis is extravasation from the vasculature. While the mechanisms regulating this process are unclear, the endothelium is known to play an important role, acting as either a barrier to some cancer cells, or a promoter of invasive capability in other cancer cells including MDA-MB-231 [15], [41]. EC apoptosis and clearance from the basement membrane have previously been reported as side effects during cancer cell extravasation [10], [13]. Similarly, ovarian tumor cell spheroids displace mesothelial cells during intercalation into the submesothelial space [16]. We demonstrate that in our *in vitro* model, most metastatic breast cancer cells clear a space in and appear to become part of the endothelium (without forming VE-cadherin dependent adherens junctions with the ECs), in a process we term “incorporation.” We were able to observe this process for multiple metastatic cancer lines, including MDA-MB-231 breast cancer cells, A375 melanoma cells, and SW1990 pancreatic cells. Notably, this process occurs independently of the activation status of the endothelium or subendothelial substrate stiffness for some cell types, even though these are both important regulators of leukocyte transmigration [19], [30].

Our data indicates that incorporation into the endothelium represents the first step in extravasation. Thus, we suggest that cancer cell transmigration is a two-step process in which the cells must first incorporate into the endothelium before completing the second step of migration beneath the endothelium and into the basement membrane or even continuing into the basement membrane without migrating beneath the ECs. Indeed, partial retraction of the ECs and incorporation of tumor cells has been observed *in vivo* [42], [43] and for mesenchymal stem cells (MSCs) [44], [45]. However, previous studies with MSCs have shown that this step depends heavily on the activation status of the endothelium and the use of VCAM-1 to facilitate transmigration [44]–[46]. In our work, which is relevant for low-flow conditions such as in the capillaries, we found that incorporation occurs independently of the activation status of the endothelium for breast cancer cells and melanoma cells. In contrast, studies have shown that fibrosarcoma cells displayed increased intravasation rates into an inflamed endothelium [47] and our results indicate that SW1990 pancreatic cells increased incorporation into the endothelium following endothelial activation. Breast tumor cells lack β2 integrins, which are a ligand for ICAM-1 on the endothelium [48]. Therefore, it is not unexpected that endothelial activation does not affect breast cancer cell incorporation into the endothelium. Following activation of the endothelium, melanoma cells also did not show an increase of incorporation fraction. It has been shown that the co-culture of melanoma cells with ECs leads to LFA-1/ICAM-1 receptor coupling during transmigration [49]. Likely, melanoma cells induce ICAM-1 expression on untreated ECs and use the LFA-ICAM-1 receptor couple to incorporate in the endothelium. Based on this previous study, it is not unexpected that melanoma cell incorporation into the endothelium does not depend on EC activation. SW1990 cells on the other hand, have been shown to increase adhesion to mesothelial cells following incubation with TNF-α [50]. This agrees with the results that we observed in this study here, though further study is necessary to dissect the specific mechanisms of pancreatic cell incorporation into an activated EC monolayer. Additionally, tumor cells are capable of attaching to leukocytes by expressing ICAM-1. The leukocytes then act as a linker, connecting the tumor cells to the endothelium, and enabling firm adhesion and subsequent transmigration [51]. *In vivo*, cancer cells may use this mechanism to localize to sites of inflammation or infection.

Additionally, we observed that tumor cell incorporation does not depend on the subendothelial substrate stiffness. Subendothelial substrate stiffness varies between homeostasis and pathological disease and likely also depends on vasculature location. When plated on stiffer substrates *in vitro*, the endothelium has a hyper-contractile phenotype that leads to intercellular gap formation [19], [27]. Thus, it would be expected that cancer cells would transmigrate more on stiffer substrates. However, despite these observations, the cumulative fraction of tumor cells that incorporates remains approximately the same independently of subendothelial substrate stiffness and the subsequent hyper-contractility of the EC monolayer for the melanoma cells and breast cancer cell lines. This indicates that the tumor cells do not take advantage of the hyper-permeability of the endothelium. However, as they migrate beyond the vasculature and into tissues, matrix stiffness may become an important modulator of metastasis; indeed, previous reports have indicated that the matrix stiffness governs 3D cancer cell migration [52]. The pancreatic cells displayed a different behavior when they incorporated in EC monolayers with varied subendothelial substrate stiffness. They displayed similar rates and fraction incorporations on soft (0.87 kPa) and intermediate (280 kPa) substrates. On very stiff 50 GPa substrates, incorporation was reduced. The microenvironmental stiffness's that cells feel *in vivo* are between 10 Pa-10000 Pa [53]. 50 GPa is well outside the physiological range, so it is possible that the cells altered their incorporation behavior based on the very stiff substrate. Elucidation of why pancreatic cells incorporated less onto very stiff subendothelial substrates would require further study.

Stephan Paget's ‘seed and soil hypothesis’ has been used to explain the non-random patterns of metastasis formation to visceral organs. Certain tumor cells (the seed) have a propensity to metastasize to particular organs (the soil) in the body. Based on our results, subendothelial substrate stiffness does not play a role in determining where cancer cells initially exit the blood stream. Furthermore, our work suggests that ligands that are upregulated during inflammation do not play a role in determining where cancer cells exit the blood stream, at least in vasculature with low shear stress rates.

Although all the metastatic tumor cells observed in this study incorporated into the endothelium, there was a significant difference between the fractions of incorporation. A375 melanoma cells incorporated into the endothelium significantly more than MDA-MB-231 cells and SW1990 cells and their incorporation dynamics closely resembled that of native ECs spreading into a monolayer. SW1990 tumor cells on the other hand, incorporated at a significantly lower rate. There are multiple factors that can account for these differences. It is likely that each type of tumor cells use distinct set of adhesion molecules and signaling pathways. Furthermore, cancer cell invasion behavior has been shown to be cancer cell specific. For example, MDA-MB-231 cells have been shown to increase their invasion into a 3D matrix in the presence of an endothelium while pancreatic cells invasion into a 3D matrix decreases in the presence of an endothelium [15]. Thus, our results are not surprising as there are multiple factors that can affect cancer cell transmigration and invasion, and these are likely cell-specific.

During incorporation, ECs do not express VE-cadherin along borders with cancer cells, but continue to express VE-cadherin with neighboring ECs. VE-cadherin was significantly restructured during the incorporation process, indicating that the addition of tumor cells disrupted the integrity of the monolayer. Additionally, this disruption may provide an easily accessible route for other incorporating cancer cells. Incorporation initiated by creating a small hole in the VE-cadherin between ECs. As incorporation progressed, the hole became much larger, creating a void for the cancer cell to spread onto the underlying fibronectin-coated matrix beneath. These results are consistent with others that have shown that during leukocyte transendothelial migration, a transient gap forms between the ECs, allowing the leukocyte to migrate through, before closing the VE-cadherin junctions [26], [54]. It remains to be seen whether the endothelium would reform junctions as the cancer cell completes extravasation. From our work, it is evident that the incorporation process damages the endothelium since tumor cells physically displace and/or detach ECs during incorporation. Thus, tumor cells damage the endothelium during incorporation, whereas leukocytes and even MSCs appear to leave the endothelium intact in most cases.

Elucidating the mechanistic underpinning for cancer cell diapedesis will be critical in order to understand cancer metastasis and its progression. Significantly, our results show that cancer cell transmigration includes an additional step, incorporation into the endothelium, and that this process occurs independently of inflammation and subendothelial substrate stiffness for some cancer types. Out of the cell lines tested, pancreatic cells displayed differences upon endothelium activation and showed a decreased fraction of incorporation on very stiff substrates. While here we specifically designed our *in vitro* model to focus on the early stages of cancer cell extravasation, future studies could be aimed at investigating subsequent stages of extravasation by characterizing cancer cell incorporation into an EC monolayer on a 3D matrix through which the tumor cells can penetrate.

## Supporting Information

Figure S1 (A) Cumulative incorporation of A375 melanoma cells into untreated and TNF-α treated endothelium after 15 hours. Data points represent mean ± SEM for at least 3 independent experiments (N>20 cells for each experiment). (B) Final fraction of incorporation after 15 hours for untreated and TNF-α treated endothelium. Data is not significant (n.s.) (P>0.05).
(TIF)

Figure S2 (A) Cumulative incorporation of SW1990 cells into untreated and TNF-α treated endothelium after 15 hours. Data points represent mean ± SEM for at least 3 independent experiments (N>20 cells for each experiment). (B) Final fraction of incorporation after 15 hours for untreated and TNF-α treated endothelium. (*) indicates significance when compared to untreated ECs (P<0.05).
(TIF)

Figure S3 (A) Cumulative fraction of A375 cells incorporated into endothelial cells on a fibronectin-coated 0.87 kPa or 280 kPa polyacrylamide gel, or glass (50 GPa). Data points represent mean ± SEM for at least 3 independent experiments (N>20 cells for each experiment). (B) Final fraction of A375 cells incorporated into the (untreated) endothelium as a function of subendothelial substrate stiffness. Bars represent mean, while error bars represent SEM of at least 3 independent experiments. P>0.05 between these values indicates there is no statistical difference (n.s.).
(TIF)

Figure S4 (A) Cumulative fraction of SW1990 cells incorporated into endothelial cells on a fibronectin-coated 0.87 kPa or 280 kPa polyacrylamide gel, or glass (50 GPa). Data points represent mean ± SEM for at least 3 independent experiments (N>20 cells for each experiment). (B) Final fraction of SW1990 cells incorporated into the (untreated) endothelium as a function of subendothelial substrate stiffness. Bars represent mean, while error bars represent SEM of at least 3 independent experiments. (*) indicates statistical difference between groups (P<0.05).
(TIF)

Movie S1 **Phase contrast image sequence of MDA-MB-231 cells incorporating into an untreated HUVEC monolayer on glass.** Scale bar is 50 µm and time after plating MDA-MB-231 cells onto the endothelium is indicated in upper right hand corner.
(AVI)

Movie S2 **Fluorescence image sequence of a DiIC_16_-labeled MDA-MB-231 cell (red) incorporating into an untreated HUVEC monolayer expressing VE-cadherin-GFP (green).** Scale bar is 25 µm and time after plating MDA-MB-231 cells onto the endothelium is indicated in upper right hand corner.
(AVI)
