# Supplementary figures and images for: VE-Cadherin-Independent Cancer Cell Incorporation into the Vascular Endothelium Precedes Transmigration
Source: PLoS One. 2014 Oct 2;9(10):e109748. doi: 10.1371/journal.pone.0109748 (PMC4183660; doi:10.1371/journal.pone.0109748)

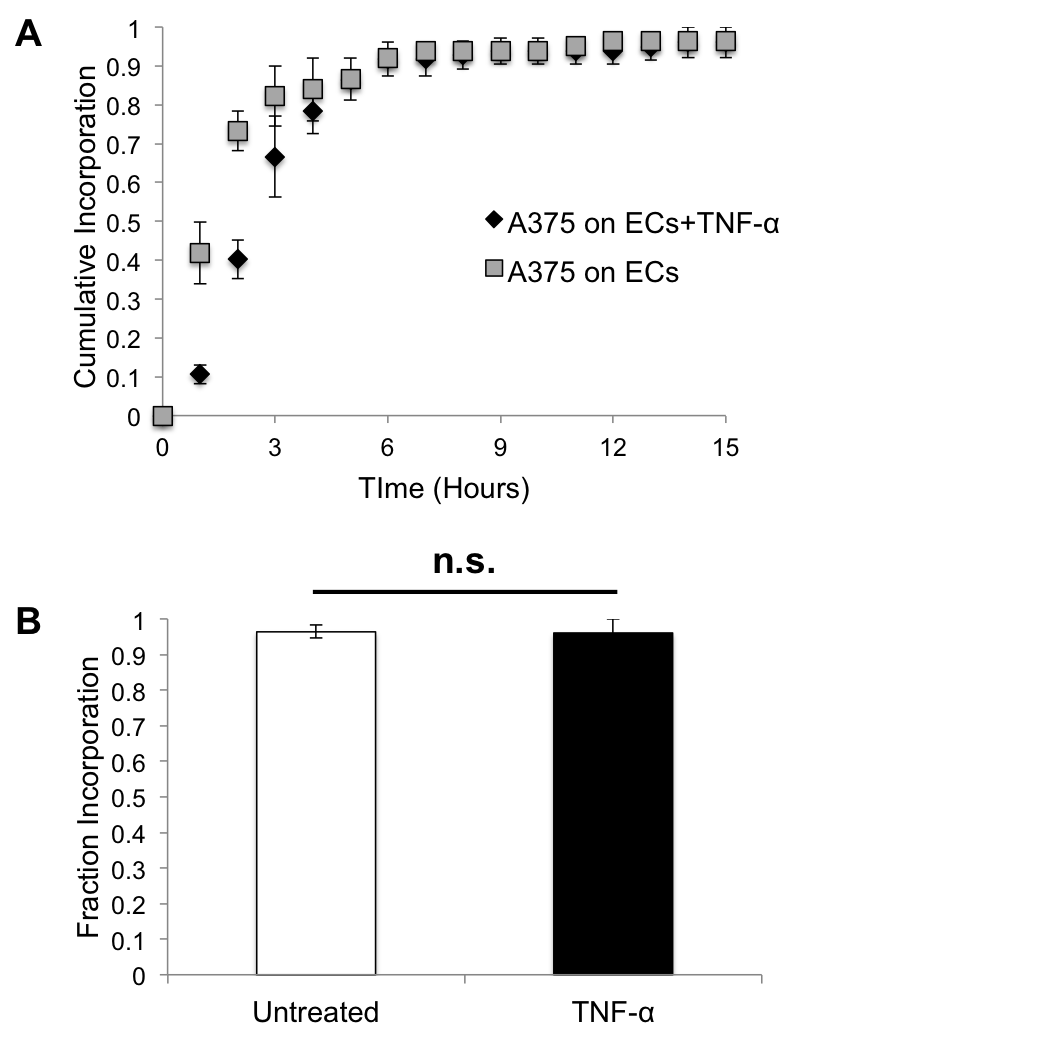

Supplement: Figure S1 — (A) Cumulative incorporation of A375 melanoma cells into untreated and TNF-α treated endothelium after 15 hours. Data points represent mean ± SEM for at least 3 independent experiments (N>20 cells for each experiment). (B) Final fraction of incorporation after 15 hours for untreated and TNF-α treated endothelium. Data is not significant (n.s.) (P>0.05). (TIF) [file pone.0109748.s001.tif]

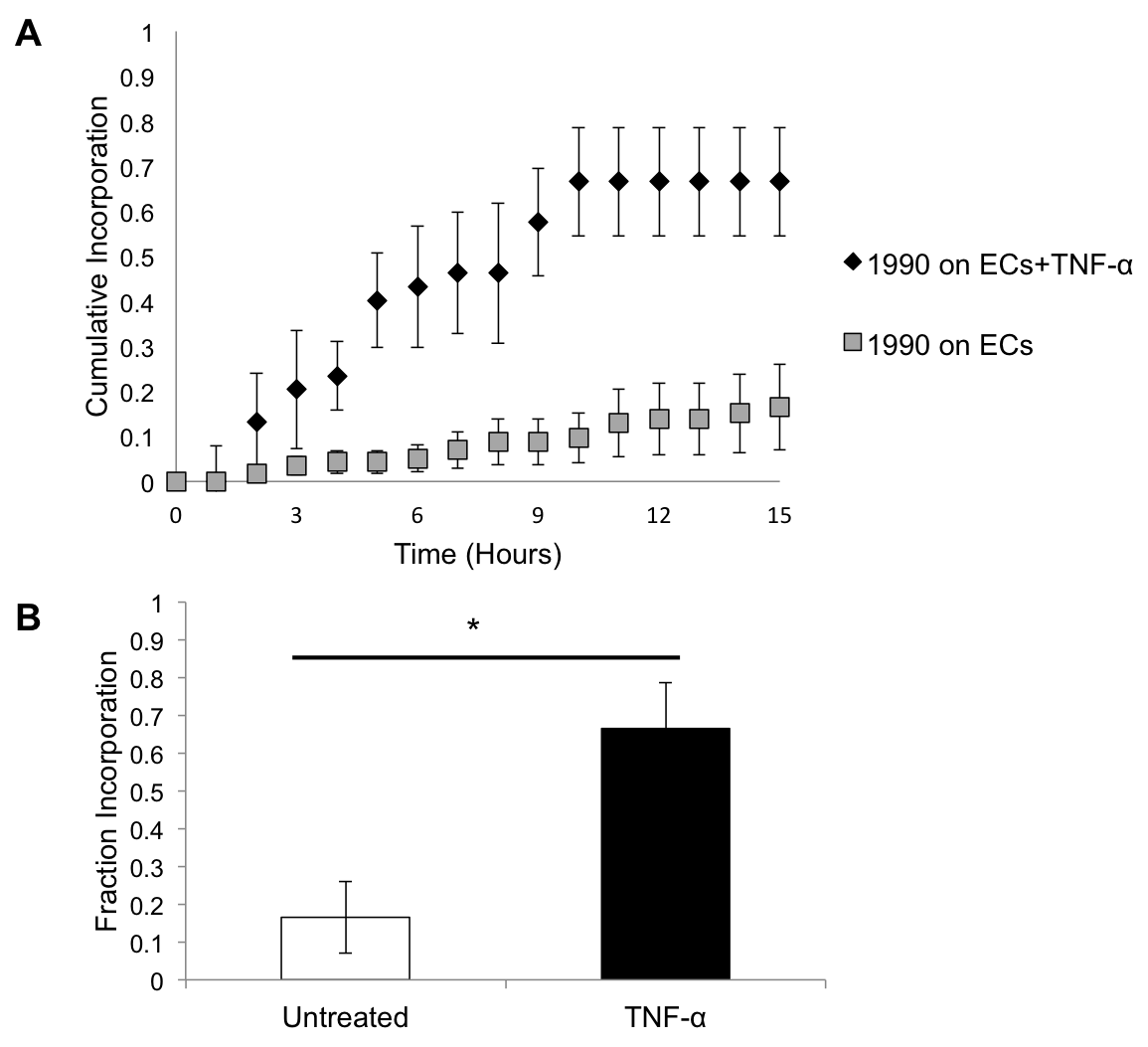

Supplement: Figure S2 — (A) Cumulative incorporation of SW1990 cells into untreated and TNF-α treated endothelium after 15 hours. Data points represent mean ± SEM for at least 3 independent experiments (N>20 cells for each experiment). (B) Final fraction of incorporation after 15 hours for untreated and TNF-α treated endothelium. (*) indicates significance when compared to untreated ECs (P<0.05). (TIF) [file pone.0109748.s002.tif]

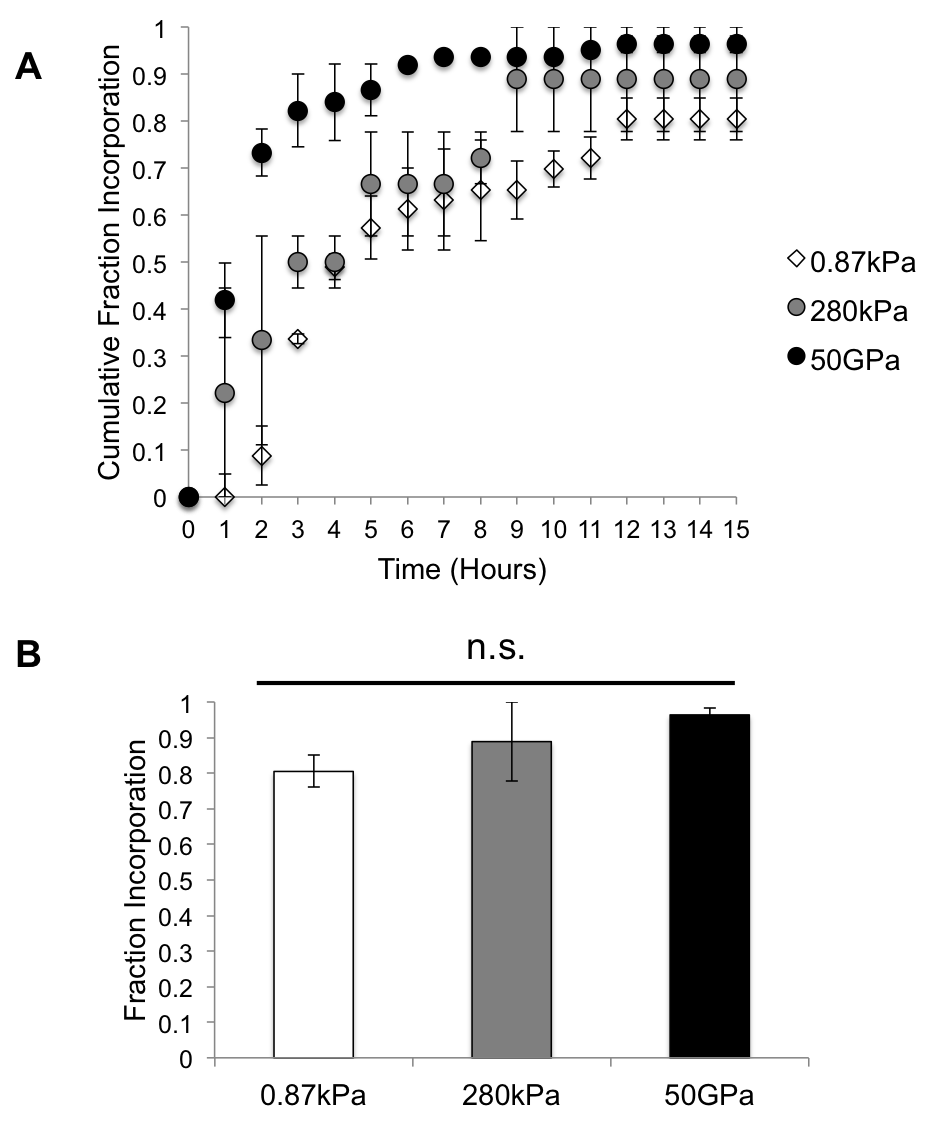

Supplement: Figure S3 — (A) Cumulative fraction of A375 cells incorporated into endothelial cells on a fibronectin-coated 0.87 kPa or 280 kPa polyacrylamide gel, or glass (50 GPa). Data points represent mean ± SEM for at least 3 independent experiments (N>20 cells for each experiment). (B) Final fraction of A375 cells incorporated into the (untreated) endothelium as a function of subendothelial substrate stiffness. Bars represent mean, while error bars represent SEM of at least 3 independent experiments. P>0.05 between these values indicates there is no statistical difference (n.s.). (TIF) [file pone.0109748.s003.tif]

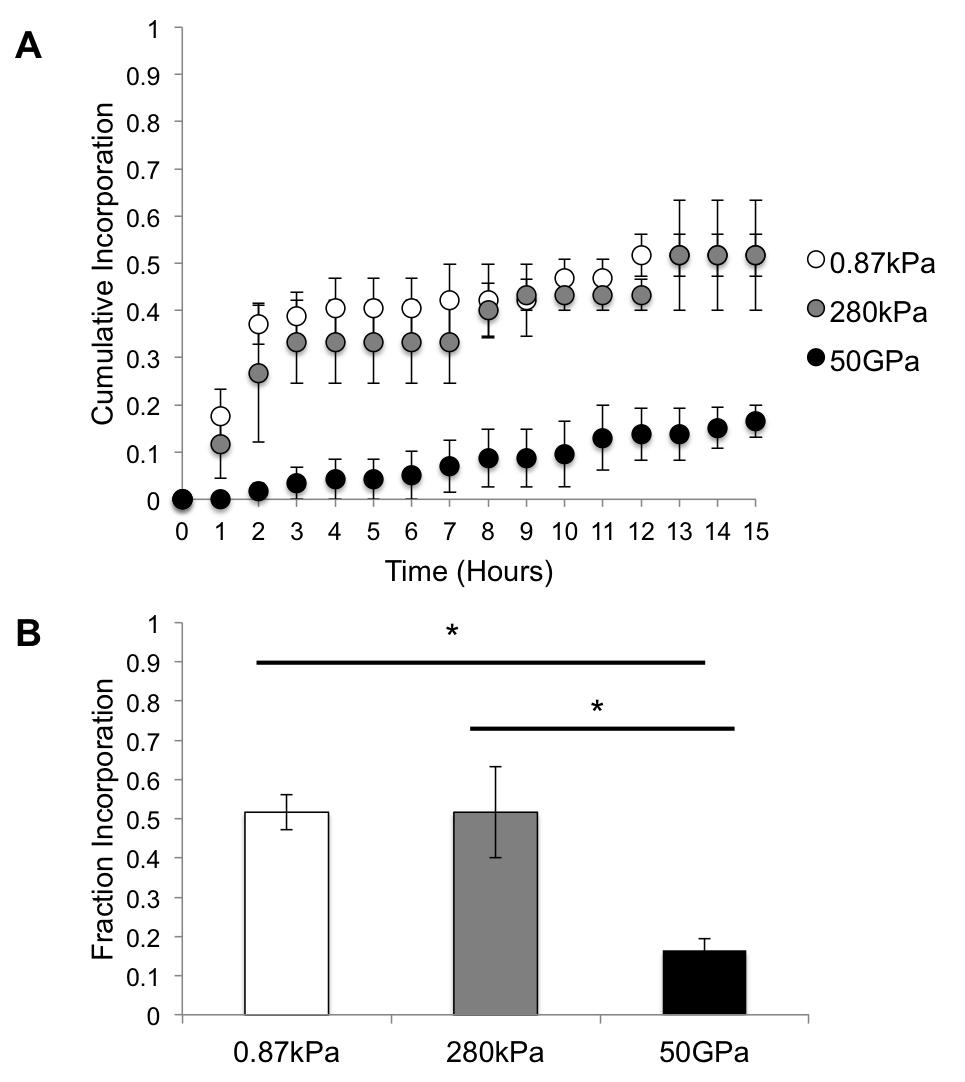

Supplement: Figure S4 — (A) Cumulative fraction of SW1990 cells incorporated into endothelial cells on a fibronectin-coated 0.87 kPa or 280 kPa polyacrylamide gel, or glass (50 GPa). Data points represent mean ± SEM for at least 3 independent experiments (N>20 cells for each experiment). (B) Final fraction of SW1990 cells incorporated into the (untreated) endothelium as a function of subendothelial substrate stiffness. Bars represent mean, while error bars represent SEM of at least 3 independent experiments. (*) indicates statistical difference between groups (P<0.05). (TIF) [file pone.0109748.s004.tif]
